# Storage and Query of Drug Knowledge Graphs Using Distributed Graph Databases: A Case Study

**DOI:** 10.3390/bioengineering12020115

**Published:** 2025-01-26

**Authors:** Xingjian Han, Yu Tian

**Affiliations:** 1 China Electronic Product Reliability and Environmental Testing Research Institute (The Fifth Electronic Research Institute of MIIT), Guangzhou 510610, China; 2 Engineering Research Center of EMR and Intelligent Expert Systems, Ministry of Education, Key Laboratory for Biomedical Engineering of Ministry of Education, College of Biomedical Engineering and Instrument Science, Zhejiang University, Hangzhou 310027, China

**Keywords:** distributed graph databases, drug knowledge graph, storage and query, real-time data retrieval

## Abstract

Background: Distributed graph databases are a promising method for storing and conducting complex pathway queries on large-scale drug knowledge graphs to support drug research. However, there is a research gap in evaluating drug knowledge graphs’ storage and query performance based on distributed graph databases. This study evaluates the feasibility and performance of distributed graph databases in managing large-scale drug knowledge graphs. Methods: First, a drug knowledge graph storage and query system is designed based on the Nebula Graph database. Second, the system’s writing and query performance is evaluated. Finally, two drug repurposing benchmarks are used to provide a more extensive and reliable assessment. Results: The performance of distributed graph databases surpasses that of single-machine databases, including data writing, regular queries, constrained queries, and concurrent queries. Additionally, the advantages of distributed graph databases in writing performance become more pronounced as the data volume increases. The query performance benefits of distributed graph databases also improve with the complexity of query tasks. The drug repurposing evaluation results show that 78.54% of the pathways are consistent with currently approved drug treatments according to repoDB. Additionally, 12 potential pathways for new drug indications are found to have literature support according to DrugRepoBank. Conclusions: The proposed system is able to construct, store, and query a large graph of multisource drug knowledge and provides reliable and explainable drug–disease paths for drug repurposing.

## 1. Introduction

Drug repurposing seeks to enhance the therapeutic value of drugs and increase the success rate of drug development. Compared to traditional drug discovery methods, drug repurposing strategies are more time- and cost-efficient, providing promising opportunities to identify new treatment options for diseases and fostering innovation in pharmaceutical research and clinical practice [1]. Drug knowledge graphs are emerging as powerful tools to support drug repurposing. These graphs comprise multisource knowledge entities, including genomics, proteomics, drug chemical properties, and clinical trial data, which are organized into a comprehensive knowledge network with various semantic relationships [2]. By semantically querying and reasoning about relationships among drugs, diseases, genes, and targets, it is possible to discover new relationships for potential treatments and adverse event warnings [3], acting as a powerful tool for drug research.

To support drug repurposing and related drug research, drug knowledge graphs require the capability to efficiently perform multi-hop queries in a large volume of knowledge entities and relationships. A multi-hop query involves tracing multi-step relationship paths in the knowledge graph to find nodes (entities) indirectly related to an initial node. In drug research, the multi-hop query finds indirect relationships between drugs and diseases through multiple intermediate entities like genes, proteins, targets, or metabolites. The multi-hop pathways may reveal implicit pharmacological mechanisms that indicate potential disease therapies or adverse events. This provides efficient and explainable candidates for drug development and safety validation.

The performance of multi-hop queries is essential for drug repurposing and is mainly affected by factors such as data volume, query complexity, and storage methods. Drug knowledge graphs typically contain a large number of entities, relationships, and properties. Multi-hop queries need to traverse multiple entities and relationships. With each additional hop, the number of possible paths increases exponentially, leading to a significant increase in query complexity. Thus, a large drug knowledge graph requires tremendous query time to find multi-hop pathways. Moreover, large-scale knowledge graphs and complex queries require substantial memory, and insufficient memory can lead to a decline in query performance.

The storage method of knowledge graphs affects the performance of multi-hop queries on large graph datasets. Current research on the efficiency of representative databases in supporting typical knowledge graph queries remains limited. Salehpour [4] et al. evaluated the performance of row-store Virtuoso, column-store Virtuoso, Blazegraph, and MongoDB using well-established benchmark datasets and queries. Their findings revealed that no single database consistently outperformed others across all benchmark scenarios, emphasizing the need to tailor database selection to the specific query types. Thus, the proper selection of storage system is crucial for drug repurposing in large drug knowledge graphs. Knowledge graphs are mainly stored by resource description framework (RDF) triple stores, relational databases, and graph databases. Triple stores using the RDF format consume significant storage space and lack corresponding query engines [5]. Complex RDF queries demand large memory spaces and are not time efficient. Relational databases do not support real-time queries for highly relevant relationships well, resulting in a lower query efficiency [6]. Graph databases have become the mainstream storage method for knowledge graphs due to flexible data structure, efficient graph query performance, and more intuitive knowledge representation that aligns with human cognition [7]. However, the drug knowledge graph possesses unique characteristics such as a large volume of entities, properties, and complex relationship depths, making it difficult for a centralized graph database to support sub-second retrieval of deep correlation relationships.

Distributed graph databases provide a promising solution for efficient storage and query of large-scale drug knowledge graphs by sharding and parallel processing [8]. However, the complexity of multi-hop queries can still lead to inefficiency due to the distribution of knowledge nodes, cross-system communication, network latency, resource scheduling, and query strategy. There remains a research gap in the storage and query performance evaluation of drug knowledge graphs based on distributed graph databases, especially regarding their application in real-world healthcare environments. Further studies are needed to assess the feasibility and performance of these methods.

In this study, we aim to evaluate distributed graph databases in terms of drug knowledge graph storage and application, with a focus on supporting drug repurposing efforts. Specifically, we design a drug knowledge graph storage and query system based on the Nebula Graph distributed graph database [9] for drug repurposing. We construct a large-scale drug knowledge graph using multisource knowledge entities to evaluate the performance of the distributed knowledge graph system, validating the performance of constrained, multi-hop queries and graph storage. Finally, we use two drug repurposing benchmarks to provide a more extensive and reliable assessment.

## 2. Materials and Methods

### 2.1. System Architecture

**Figure 1:**
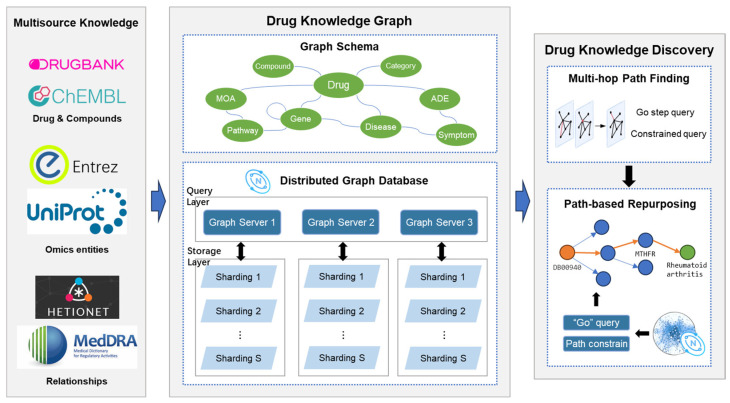
Architecture of drug knowledge graph storage and query system [10].

The overall architecture of the drug knowledge graph storage and query system constructed in this study is shown in Figure 1. The drug knowledge graph consisted of multisource knowledge, including drug compounds, omics, diseases, pathways, and their relationships. The entities and relationships were standardized with certain labels, properties, and annotations. A graph schema connected multisource knowledge entities, forming a global network of drug-related resources. To efficiently store and utilize this knowledge and maintain scalability, a distributed graph architecture was implemented for knowledge graph storage and query [10]. The system was deployed on N servers, each with S slices for data storage. The graph query layer had corresponding N graph services for knowledge graph queries which interacted with both the data storage layer and the drug knowledge discovery. The storage layer used multiple servers with multiple data sharding to enhance query efficiency for multisource large-scale knowledge graphs. Drug knowledge discovery was the application of the drug knowledge graph to perform graph browsing and multi-hop queries. The Nebula Graph’s built-in browser empowers graphical usage with pre-defined nGQL query statements. The multi-hop query was achieved by constrained queries and the Nebula Graph’s Go step query in a distributed graph database setting.

In this implementation, we chose to deploy the graph database on three servers. These distributed servers were managed in a cluster mode, ensuring unified data management and high availability. Multiple data slices were stored on each server, with the number of slices set to five times the number of hard disks in the cluster. Additionally, to prevent service interruption in the event of server failure or downtime, each complete dataset was backed up across these three servers. When the system faced high concurrent query requests, these three servers worked together to share the query load, thus ensuring query efficiency. Each server simultaneously deployed data storage services and computation services.

Nebula Graph uses sharding to distribute data evenly across storage nodes and consistent hashing to minimize data skew. It improves query efficiency by storing all tags, outgoing edges, and incoming edges of a node in the same shard. Query routing is managed by the meta service, which directs requests to the most appropriate node, optimizing performance. For fault tolerance, Nebula Graph relies on the Raft consensus protocol and ensures data consistency across replicas through log replication. Even during node failures, other replicas continue to provide services, maintaining high availability. Once a failed node is recovered, the meta service automatically synchronizes the missing data and restores their state, ensuring data integrity. In addition, Raft supports automatic leader election, allowing the system to recover quickly and remain stable during failures.

### 2.2. Data Storage and Retrieval Methods

**Figure 2:**
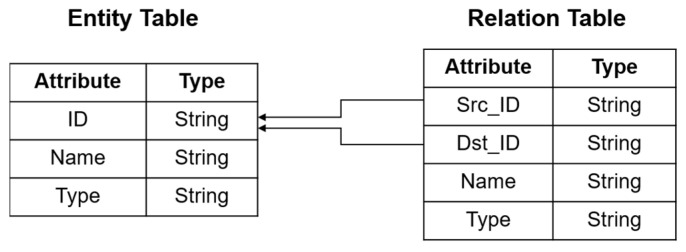
Drug knowledge graph original data storage schema.

**Figure 3:**
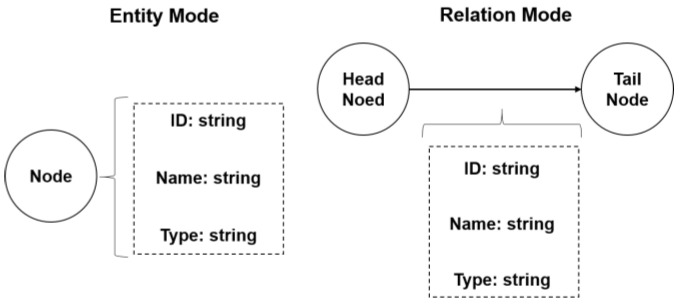
Drug knowledge graph data storage schema.

**Figure 4:**
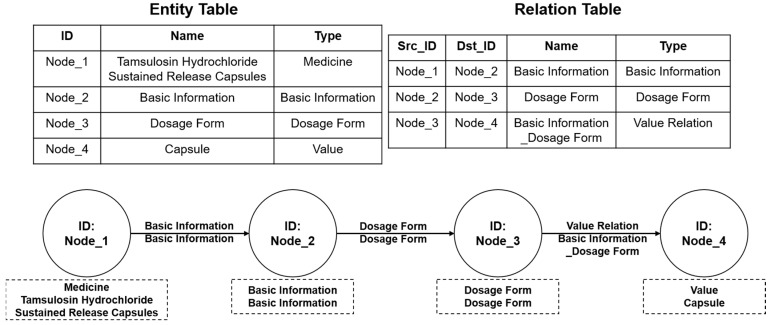
Drug knowledge graph data example.

In this system, drug knowledge graph data were extracted from various heterogeneous medical knowledge sources, such as drug instructions, adverse drug reaction reports, medical literature, and clinical guidelines. The original drug knowledge graph data were stored according to the structure shown in Figure 2. Next, the drug knowledge graph pattern was constructed. Nebula Graph uses a directed property graph for modeling. The first step was to build the entity pattern. All attribute fields were established based on the original data storage table, and all attributes in the table were mapped to the entity’s attributes. In this study, all entities, entity relationships, and attribute values were abstracted as entity nodes and stored in the entity pattern. The second step was to build the relationship pattern. In Nebula Graph, edges are directed, and each edge represents a relationship from a head entity to a tail entity. The head and tail entities come from the nodes in the entity pattern. The head and tail nodes, pointing information, and attribute information of the relationship can be extracted from the relationship table in the original data. The constructed entities and their relations are shown in Figure 3. Finally, data extraction, transformation, and loading were performed. Compliant nodes and edges were constructed according to Nebula Graph specifications and loaded into the database. Figure 4 provides an example of the data before and after importing them into the database.

### 2.3. System Evaluations

In order to evaluate the data writing and query performance of the drug knowledge graph based on the distributed Nebula Graph database, particularly the performance of multi-hop queries, this study conducted performance tests using real drug knowledge graph data and compared them with the performance under a centralized deployment.

The data used for performance evaluation came from multiple heterogeneous medical knowledge sources. The drug knowledge information selected in this study was based on large standard public datasets such as Drugbank, ATC, SNOMED, MedDRA, ChEMBL, Entrez, Uberon, UniProt, SMPDB, and Hetionet. It covered various aspects of knowledge, including drugs, diseases, molecules, adverse drug reactions, proteins, anatomy, pathways, drug components, and contraindications. A total of 7378 drugs were covered, involving 14 categories of entities, with 182,864 entity nodes and 100 types of relationships, amounting to 2,613,643 relationship nodes. This drug knowledge graph has extensive applicability, operates on a large scale, and is highly representative of real-world medical scenarios.

The system evaluated and recorded the time consumption of Nebula Graph in both centralized and distributed modes under different data write volumes. In each experiment, existing data were cleared before importing new data. The amount of data inserted into the database started at 1 million nodes, increasing by 1 million nodes each time, until it reached 15 million nodes.

Meanwhile, the average time consumption of Nebula Graph was tested during 1000 data queries, with the query tasks conducted in both centralized and distributed modes. The drug knowledge graph used in the test contained approximately 18 million nodes and 29 million relationships. Each query was flexibly designed based on actual medical knowledge query needs, with the queries categorized into regular queries and constrained queries. Regular queries were further divided into single-hop path queries and multi-hop path queries, with the longest path in the multi-hop queries being five hops. Finally, we tested the performance of multi-hop queries in concurrent scenarios, including single-hop and multi-hop queries.

Constrained multi-hop queries are similar to the query requirements in drug repurposing scenarios. Statistical differences in writing and query times between centralized and distributed modes were determined using statistical tests. If the data followed a normal distribution, a paired *t*-test was used; if the data did not follow a normal distribution, the Wilcoxon signed-rank test was used.

We conducted two-hop path-based queries with constrained entity labels and relationship labels to investigate the drug repurposing scenarios. The query paths began with drug ingredient entities and edges connecting to gene/target entities with certain relation types. The query then explored the functional relationships with disease entities for each gene or target. Represented as (vertex1:Drug)-[relation 1]-(vertex2:Gene/Target)-[relation 2]-(vertex3:Disease), the two-hop query aimed to identify drug–disease relationships by distributed architecture for efficiency.

To assess accurately queried drug–disease paths and potential new pathways for drug repurposing, two benchmark drug-repurposing datasets were used to indicate valid pathways. The repoDB is a gold standard database with approved and failed drugs according to drug labels and clinical trials [11]. This dataset was used to verify that the two-hop paths were consistent with currently approved drug treatments, as well as terminated, suspended, and withdrawn drug studies. This database works as a baseline evaluation for existing information. The DrugRepoBank [12] is a database encompassing 169 repurposed drugs from 134 articles. This dataset was used to match new repurposing pathways that have been reported in articles with in vitro studies. This database evaluated the potential of the proposed system to find new pathways that were assessed by research.

## 3. Results

### 3.1. System Implementations

The system was deployed on three identically configured servers, and the specific configuration of the servers is shown in Table 1. The Nebula Graph database was installed on the servers, and the configuration of each server was adjusted to enable communication between them. In the distributed deployment plan of this study, the graph database was deployed across three servers. The specific deployment information is shown in Table 2. Specifically, Server A initiated one “metad” process, one “graphd” process, and one “storaged” process; Server B started one “graphd” process and one “storage” process; and Server C started one “graphd” process and one “storaged” process.

The system provides the interactive interface for searching the central node. After entering the concept to be searched (i.e., the central node of the drug knowledge graph to be queried), selecting the display level, and clicking the search button, a list of similar concepts is displayed. By selecting the matching concept, a knowledge graph centered on the search concept with the selected display level as the depth is generated. Hovering over a node displays its specific name. When clicking on a leaf node, the system generates a new graph centered on that leaf node with a depth level of two and links it to the original graph information. For non-leaf nodes, clicking collapses their child nodes. After selecting the central node, the system automatically calculates and generates the module to display this graph. Clicking on a node allows you to expand or collapse its child nodes.

### 3.2. System Evaluation Results

**Figure 5:**
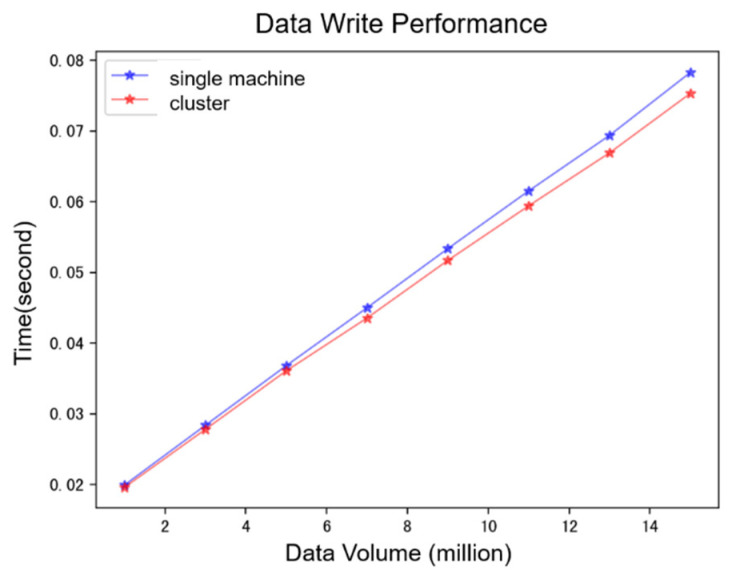
Database write performance.

The database write performance test results are presented in Figure 5. As illustrated, the time consumption in both centralized (single-node) and distributed (cluster) modes exhibited a linear increase as the data write volume grew. Notably, the writing time in the cluster mode outperformed that of the single-node mode (*p* = 0.002), with the performance gap between the two modes widening as the data volume increased. This highlights the growing efficiency advantage of distributed cluster writes over time.

**Figure 6:**
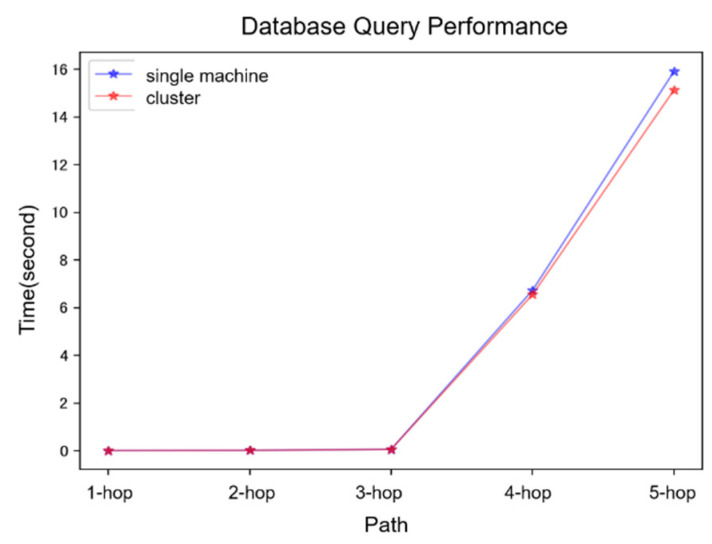
Regular query performance.

**Figure 7:**
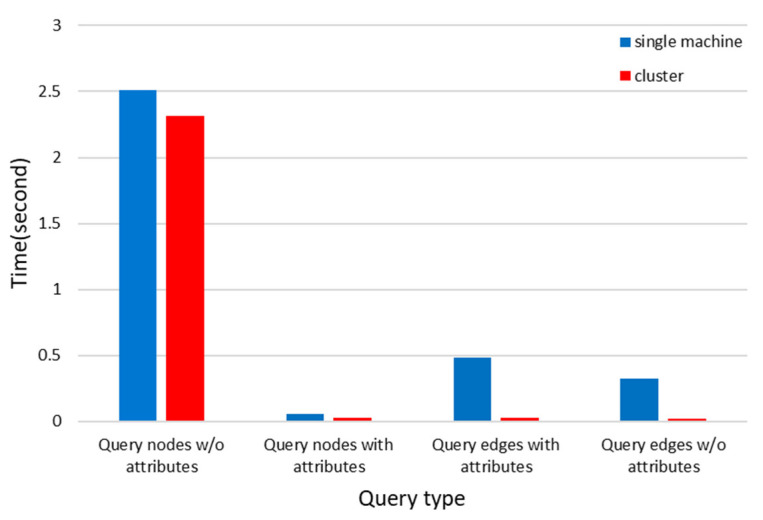
Constrained query performance.

The results of the regular query performance are depicted in Figure 6. The queries included both single-hop and multi-hop paths. For queries involving up to three hops, both single-machine and cluster modes exhibited relatively short query times with minimal differences between them. In contrast, for four-hop and five-hop queries, there was a substantial increase in query time, with cluster queries demonstrating a slight performance advantage over single-machine queries (*p* = 0.313). The performance test results of constrained queries are shown in Figure 7. Whether querying nodes or edges, the efficiency of cluster queries was superior to that of single-machine queries (*p* < 0.001).

The results of the constrained two-hop path queries for drug repurposing are presented in Table 3. The table summarizes all valid two-hop paths, from drug ingredient entities to disease entities with constrained path conditions. There were nine distinct two-hop paths among drugs, genes/targets, and diseases. The query was executed in cluster mode, yielding a total of 119,730 paths, each indicating a drug–disease relationship by a specific gene/target. From a drug entity perspective, the average query time to find all valid two-hop paths of a drug was 0.0009 s, with a total query time of 108.24 s to screen all drug entities. The results demonstrate the feasibility and efficiency of distributed knowledge-graph-driven drug repurposing research.

Table 4 illustrates the evaluation result of two-hop queries according to repoDB. The evaluation knowledge graph matched 318 drugs with 44 diseases with repoDB, resulting in 424 total drug–disease paths. Among them, 333 paths (78.54%) are currently approved treatments. Note that the two-hop query demands a drug–gene/target–disease path; therefore, not all drug–disease pairs are able to match.

The evaluation of potential new drug repurposing paths according to DrugRepoBank is illustrated in Table 5. The benchmark database matched 12 studies indicating potential new treatments for these drugs. The matched drug–disease pairs were reported by articles with in vitro studies. For example, sildenafil was used, initially, for erectile dysfunction. The query indicated that sildenafil targets CYP3A4, which helps treat lung cancer. Current evidence already indicates the anticancer properties of sildenafil, such properties strongly warranting clinical investigation. Clenbuterol is used for treating acute asthma through the β2-adrenergic receptor (related to ADRB2). The target can also increase the proliferation rate of neural progenitor cells, a phenomenon which may help treat Down syndrome. Bromocriptine was initially used for Parkinson’s disease. The query indicated that Bromocriptine functions on ADRA2A (α_2A_ adrenergic receptor), which is related to diabetes through the regulation of pancreatic hormone secretion. The literature-supported pathways demonstrated that the proposed system was able to perform multi-hop queries for drug repurposing scenarios with high efficiency. Because drug repurposing requires a combined search across multiple knowledge sources, the distributed knowledge graph structure is helpful in such settings.

**Figure 8:**
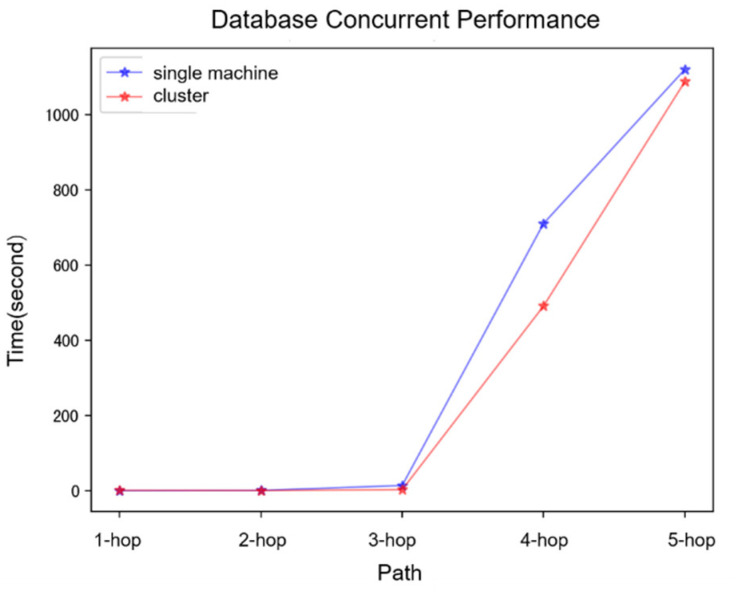
Concurrent query performance.

The results of the concurrent query performance for the database are presented in Figure 8. During testing, the concurrency level was set to 10. For three-hop queries, the efficiency of concurrent queries in single-machine and cluster modes was essentially comparable, likely due to the single machine’s adequate performance in handling 10 concurrent requests. However, as the number of hops increased to four and five, the efficiency of concurrent queries on a single machine declined relative to that of the cluster mode (*p* = 0.016).

## 4. Discussion

This study designed and implemented a drug knowledge graph storage and query system based on the Nebula Graph database and evaluated its performance. To our knowledge, this is the first application of a distributed graph database in a medical context for drug knowledge graphs, and we assessed its performance to establish feasibility and performance benchmarks for such databases in the medical field. Nebula Graph was selected for its ability to efficiently handle large-scale, distributed graph data, an ability which is crucial for managing the complexity of drug knowledge graphs. Its architecture supports high-performance querying, fault tolerance, and scalability, making it an ideal choice for handling the multi-hop queries required in drug repurposing applications [9]. Additionally, Nebula Graph’s open-source nature and active community support were key factors in our decision.

The results show that the performance of the distributed graph database exceeds that of single-machine databases in data writing, regular queries, constraint queries, and concurrent queries. Additionally, as the volume of the data increases, the advantages of the distributed graph database in writing performance become more pronounced, highlighting the effectiveness of distributed architectures in large-scale data storage scenarios. As the complexity of query tasks increases, the performance advantages of the distributed graph database also improve. Our findings also indicate that, in complex query paths, the distributed graph database maintains its performance advantage over single-machine databases despite frequent inter-node communication and dynamic resource scheduling. However, the increased query complexity actually results in longer query times in the Nebula Graph database. In scenarios with a higher number of nodes, relationship types, and hops, the query time becomes longer. Multi-hop queries require traversing more nodes and edges, a phenomenon which can significantly increase query time, especially when dealing with large graph datasets. Strategies to mitigate these challenges should be further studied, such as query optimization techniques and improvements in computational efficiency.

The drug knowledge graph deployed with the Nebula Graph database in a distributed setting can effectively support research in drug repurposing, adverse reaction warning, and pathway discovery. The two-hop drug repurposing pathway queries identified 119,730 paths within a reasonable time. Literature reviews indicated that the query was able to find pathways for new indications of certain drugs. The knowledge graph-based query provides explainable repurposing targets and helps clinical research with specific genes/targets. Given that the drug knowledge graph involves 182,864 entities and 2,613,643 relationships with multi-dimensional properties, the Nebula Graph excels at managing and processing these heterogeneous data, efficiently handling the complex relationships among entities such as diseases, genes, proteins, and drugs, making it particularly suited for multi-hop queries to discover new pathways. By distributing storage and query tasks across multiple nodes, Nebula Graph offers enhanced scalability, performance, and fault tolerance, enabling researchers to explore complex, multi-step connections within the knowledge graph. Considering that the scale and complexity can impact query times and reduce research efficiency, the distributed architecture ensures that researchers can quickly access relevant information, thereby accelerating new pathway discoveries and helping drug development and safety research.

## 5. Conclusions

In summary, the proposed system was able to construct, store, and query a large graph of multisource drug knowledge and provided reliable and explainable drug–disease paths for drug repurposing. The performance was better than that resulting from using a non-distributed architecture. The multi-hop repurposing query was tested both on currently approved drugs and new literature-supported treatments. This study provides a foundational framework for distributed graph database applications in drug research, paving the way for advancements in biomedical informatics.

## Figures and Tables

**Table 1:** Server configuration information.

Configuration Information	Details
CPU cores	4
Memory	32 G
Hard disk	100 G
Operating system	CentOS 7

**Table 2:** Configuration information of graph database nodes.

Machine Name	Number of Metad Processes	Number of Storaged Processes	Number of Graphd Processes
Server A	1	1	1
Server B	-	1	1
Server C	-	1	1

**Table 3:** Results of two-hop drug repurposing queries.

Path Type	Relation 1	Relation 2	Count of Paths	Count of Drugs	Count of Conditions	Query Time (Second)
1	Upregulates	Upregulates	11,591	578	36	10.58
2	Upregulates	Associates	26,348	583	99	22.40
3	Upregulates	Downregulates	9570	551	36	8.66
4	Downregulates	Upregulates	15,450	610	37	13.97
5	Downregulates	Associates	19,866	563	97	17.96
6	Downregulates	Downregulates	10,029	600	33	10.12
7	Binds	Upregulates	18,575	915	33	16.91
8	Binds	Associates	3830	1292	98	3.52
9	Binds	Downregulates	4471	974	36	4.12
Total	-	-	119,730	1353	103	108.24

**Table 4:** Evaluation summary of two-hop drug–disease pairs using repoDB.

Drug Status	Drugs	Diseases	Two-Hop Paths	Drug–Disease Pairs
repoDB matched	318	44	2303	424
Approved	266	34	1603	333 (78.54%)
Terminated	53	25	508	63 (14.86%)
Withdrawn	25	11	173	24 (5.60%)
Suspended	5	4	19	4 (0.09%)

**Table 5:** Drug–disease pairs from two-hop query results supported by the literature in DrugRepoBank.

Drug	Original Treatment	Repurposing Path	Literature
Sildenafil	Erectile dysfunction	$Sildenafil\overset{Binds}{\to }{CYP3A4}^{\;1}\overset{Assoc.}{\to }Lung\;Cancer$	[13]
Indomethacin	Rheumatoid arthritis	$Indomethacin\overset{Binds}{\to }{PPARG}^{\;2}\overset{Down\;Reg.}{\to }Liposarcoma$	[14]
Amodiaquine	Malaria	$Amodiaquine\overset{Up\;Reg.}{\to }{CASP3}^{\;3}\overset{Assoc.}{\to }{NSCLC}^{\;4}$	[15]
Simvastatin	Hyperlipidemia	$Simvastatin\overset{Up\;Reg.}{\to }{IL1B}^{\;5}\overset{Assoc.}{\to }Antibacterial$	[16]
Simvastatin	Hyperlipidemia	$Simvastatin\overset{Up\;Reg.}{\to }{HMGCR}^{\;6}\overset{Assoc.}{\to }Prostate\;cancer$	[17]
Disulfiram	Alcoholism	$Disulfiram\overset{Down\;Reg.}{\to }{PCNA}^{\;7}\overset{Assoc.}{\to }Gastric\;cancer$	[18]
Salmeterol	Asthma	$Salmeterol\overset{Up\;Reg.}{\to }{ADRB2}^{\;8}\overset{Assoc.}{\to }Down\;syndrome$	[19]
Dasatinib	Multiple myeloma	$Dasatinib\overset{Binds}{\to }{BTK}^{\;9}\overset{Up\;Reg.}{\to }B\;cell\;malignancies$	[20]
Clenbuterol	Asthma	$Clenbuterol\overset{Binds}{\to }ADRB2\overset{Assoc.}{\to }Down\;syndrome$	[19]
Niclosamide	Cestodes infection	$Niclosamide\overset{Down\;Reg.}{\to }{AKT1}^{\;10}\overset{Assoc.}{\to }{HCC}^{\;11}$	[21]
Bromocriptine	Parkison’s disease	$Bromocriptine\overset{Binds}{\to }{ADRA2A}^{\;12}\overset{Assoc.}{\to }Typ\;2\;Diabetes$	[22]
Crizotinib	Non-small-cell lung cancer	$Crizotinib\overset{Down\;Reg.}{\to }{CTPS1}^{\;13}\overset{Assoc.}{\to }Gram\;Positive\;Bacteria$	[23]

^1^ CYP3A4: 5,10-methylenetetrahydrofolate reductase. ^2^ PPARG: peroxisome proliferator activated receptor gamma gene. ^3^ CASP3: caspase-3. ^4^ NSCLC: non-small cell lung cancer. ^5^ IL1B: interleukin-1 beta gene. ^6^ HMGCR: 3-hydroxy-3-methyl-glutaryl-coenzyme A reductase. ^7^ PCNA: proliferating cell nuclear antigen. ^8^ ADRB2: adrenoceptor beta 2. ^9^ BTK: bruton tyrosine kinase. ^10^ AKT1: AKT serine/threonine kinase 1. ^11^ HCC: hepatocellular carcinoma. ^12^ ADRA2A: α2A adrenergic receptor. ^13^ CTPS1: cytidine 5-prime triphosphate synthetase 1.

## Data Availability

The data presented in this study are available on request from the corresponding author. The data are not publicly available due to privacy and ethical restrictions.
